# Evidence of Simultaneous Circulation of West Nile and Usutu Viruses in Mosquitoes Sampled in Emilia-Romagna Region (Italy) in 2009

**DOI:** 10.1371/journal.pone.0014324

**Published:** 2010-12-15

**Authors:** Mattia Calzolari, Paolo Bonilauri, Romeo Bellini, Alessandro Albieri, Francesco Defilippo, Giulia Maioli, Giorgio Galletti, Antoni Gelati, Ilaria Barbieri, Marco Tamba, Davide Lelli, Elena Carra, Paolo Cordioli, Paola Angelini, Michele Dottori

**Affiliations:** 1 Istituto Zooprofilattico Sperimentale della Lombardia e dell'Emilia Romagna “B. Ubertini” (IZSLER), Brescia, Italy; 2 Centro Agricoltura Ambiente “G. Nicoli” (CAA), Crevalcore, Italy; 3 Azienda USL Modena, Mirandola, Italy; 4 Regione Emilia-Romagna, DG Sanità e Politiche Sociali, Bologna, Italy; University of Liverpool, United Kingdom

## Abstract

**Background:**

In recent years human diseases due to mosquito-borne viruses were increasingly reported in Emilia-Romagna region (Italy), from the chikungunya virus in 2007 to the West Nile virus (WNV) in 2008. An extensive entomological survey was performed in 2009 to establish the presence and distribution of mosquito arboviruses in this region, with particular reference to flaviviruses.

**Methodology/Principal Findings:**

From May 6 to October 31, a total of 190,516 mosquitoes were sampled in georeferenced stations, grouped in 1,789 pools according date of collection, location, and species, and analyzed by reverse transcription polymerase chain reaction (RT-PCR) to detect the presence of RNA belong to *Flavivirus* genus. WNV was detected in 27 mosquito pools, producing sequences similar to those of birds and human strains obtained in 2008 outbreak, pointed out the probable virus overwintering. Isolation of WNV was achieved from one of these pools. Moreover 56 pools of mosquitoes tested positive for Usutu virus (USUV). Most PCR positive pools consisted of *Culex pipiens*, which also was the most analyzed mosquito species (81.4% of specimens); interestingly, USUV RNA was also found in two *Aedes albopictus* mosquito pools. Simultaneous circulation of WNV and USUV in the survey area was highlighted by occurrence of 8 mosquito WNV- and USUV-positive pools and by the overlaying of the viruses “hot spots”, obtained by kernel density estimation (KDE) analysis. Land use of sampled stations pointed out a higher proportion of WNV-positive *Cx. pipiens* pool in rural environments respect the provenience of total sampled pool, while the USUV-positive pools were uniformly captured in the different environments.

**Conclusions/Significance:**

Obtained data highlighting the possible role of *Cx. pipiens* mosquito as the main vector for WNV and USUV in Northern Italy, and the possible involvement of *Ae. albopictus* mosquito in USUV cycle. The described mosquito-based surveillance could constitute the foundation for a public health alert system targeting mosquito borne arboviruses.

## Introduction

West Nile virus (WNV) and Usutu virus (USUV) are mosquito-borne flaviviruses of the Japanese encephalitis antigenic complex [1]; these two viruses are also phylogenetically closely related, as shown by nucleotide sequence data [2], [3], [4].

Even if USUV ecology is less known than the WNV one, the two viruses seem to show biological cycle similarities: the principal vectors of WNV and USUV are largely ornithophilic mosquitoes, mainly of the genus *Culex*, wild birds are principal reservoirs of WNV and they are suspected of being also of USUV, both viruses can be pathogenic for these animals [5], [6], [7], [8]. Besides, migratory birds are considered to play a key role in the diffusion of the two viruses in Europe from Africa [4], [9], [10], [11], where they were discovered [12], [13].

WNV can be transmitted occasionally by mosquitoes to vertebrates other than birds; these are considered dead-end host or seem to have only a minor role in the environmental maintenance of the virus. USUV was rarely isolated from mammals; only reported isolations were from *Praomys* rat and from man [7], [14]. While the WNV risks for human health is well recognized, medical importance of USUV is not fully understood, but in summer 2009 USUV-related illness were reported in two immunecompromised patients in Emilia-Romagna region [14], [15]. Furthermore, Usutu virus was detected in serum of two organ donors tested in a retrospective WNV screening performed in Italy in 2009 [16].

The first detection of USUV in Europe dates back to 2001 in Vienna [9], where the virus caused the death of hundreds of wild birds; subsequent virological and serological surveys pointed out the presence of USUV in different European countries, including Czechland and Poland [10], Spain [17], England [18], Hungary [19], Switzerland [20] and Northern Italy [21], [22], [23].

WNV is widely distributed through Africa, Asia, Europe, and Australia, and it is also present in America, since 1999. In Europe, epidemics with tens to hundreds of human cases have been observed in southern Russia, south France, Spain, Romania and Hungary since the 1960s [10], [24]. In Italy an equine outbreak of WNV, without human clinical reported cases, occurred in 1998 in “Padule del Fucecchio”, a wetland area in the Toscana region [25]. Ten years later, in 2008, WNV re-circulated in three different Regions of Northern Italy (Emilia-Romagna, Veneto and Lombardia), causing illness in horses [26] and also 9 human cases of neuroinvasive disease [27]. The epidemic resumed in 2009 affecting a wider area and causing 16 neuroinvasive human disease cases since the end of August [27]. Moreover the evidence of seroconversion due to WNV in sentinel chickens obtained in Italy [21] and in United Kingdom [18], without contemporaneous clinical disease in animals or men, implies a silent circulation of this virus.

Detections of different human diseases due to mosquito-borne viruses in Emilia-Romagna in recent years (chikungunya virus in 2007, WNV in 2008 and USUV in 2009) strongly suggested the need for an investigation to determine the presence and geographic distribution of arboviruses in this region. For this purpose, a mosquito based survey was started in late summer 2007; this plan was implemented to cover the whole season in 2008, sampling and analyzing 47,453 specimens belonging to nine mosquito species from Emilia-Romagna [28]. The survey continued in 2009, detecting the presence of USUV besides that of WNV in tested mosquitoes. In this work, the spread of these two viruses in the survey area is described and their possible interactions investigated.

## Results

A total of 158 traps worked, with different frequency and in different periods (Table 1) collecting 190,516 mosquitoes (Table 2), of which 96,270 were obtained from traps managed by mosquito control programs, 89,457 were obtained from dedicated stations and 4,789 from extraordinary stations (Table 3).

**Table 1:** Characteristic of sampling station groups with the reference to obtained PCR-positive mosquito pools.

	Stations	First sampling	Last sampling	Collection frequency	Trap	WNV PCR/+	USUV PCR/+
Bassa Bolognese (BB)	21	6-May	8-Oct	weekly	CO_2_	6^1^	29^7^
Surveillance sample (SS)	37	12-Jun	14-Oct	monthly	CO_2_	5^2^	4^8^
Modena province (MO)	12	24-Jun	8-Oct	weekly	CO_2_	5^3^	4^9^
Lidi Ferraresi (LF)	19	4-Jun	2-Oct	weekly	CO_2_		1
Parma province (PR)	13	11-Aug	8-Oct	monthly	CO_2_	1^4^	1
Reggio province (RE)	2	28-Jun	24-Sep	weekly	CO_2_	1^5^	2
Ferrara city (FE)	13	2-Sep	18-Sep	be-weekly	CO_2_		2
Piacenza province (PC)	2	23-Jun	13-Oct	be-weekly	CO_2_		
Forlì-Cesena province (FC)	5	19-Sep	31-Oct	weekly	CO_2_		1
Extraordinary samples (EX)	34	25-Jul	13-Oct	single	GT/CO_2_	9^6^	12
Total	158	6-May	13-Oct				

Trap model abbreviations: CO_2_, modified CDC traps baited by CO_2_; GT, gravid traps. GenBank accession numbers:

1 HM138728, HM138729, HM138730, HM138731;

2 HM138725, HM138726, HM138727, HM138732, HM138734;

3 HM138724, HM138737, HM138738, HM138739, HM138740;

4 HM138743;

5 HM138745;

6 HM138733, HM138735, HM138736, HM138741, HM138742, HM138744, HM138746, HM138747;

7 HM138707, HM138708, HM138710, HM138711, HM138712, HM138713, HM138714, HM138716, HM138717, HM138718;

8 HM138715;

9 HM138709.

**Table 2:** Total number of specimens, PCR pools and PCR-positive pools collected for every mosquito species in 2009 survey.

Mosquito species	Specimens (%)	Pool No	WNV PCR/+	USUV PCR/+
*Ae. albopictus*	1,227 (0.6)	108		2
*Ae. caspius*	29,283 (15.4)	314		
*Ae. detritus*	5 (<0.1)	2		
*Ae. dorsalis*	13 (<0.1)	1		
*Ae. geniculatus*	8 (<0.1)	3		
*Ae. vexans*	4,597 (2.4)	60		
*An. maculipennis*	82 (<0.1)	14		
*An. plumbeus*	2 (<0.1)	2		
*Cx. modestus*	246 (0.1)	26		
*Cx. pipiens*	155,053 (81.4)	1,259	27	54
Total	190,516	1,789	27	56

**Table 3:** Number of specimens and PCR pools collected for every mosquito species for the different station groups.

	Mosq. Abundance St.s				Survey Stations														EX	
	BB		LF		SS		MO		PR		RE		FE City		PC		FC			
Species	N	Ps	N	Ps	N	Ps	N	Ps	N	Ps	N	Ps	N	Ps	N	Ps	N	Ps	N	Ps
*Ae.albopictus*	169	13			135	7	189	19	219	4	4	3	257	24	58	11	11	4	185	23
*Ae.caspius*	1,627	49	5,472	67	5,170	57	16,912	120	11	1	7	2	3	3	2	1	14	5	65	9
*Ae.detritus*																			5	2
*Ae.dorsalis*							13	1												
*Ae.geniculatus*							6	2			2	1								
*Ae.vexans*					86	4	4,090	41	363	3	2	1			1	1	11	2	44	8
*An.maculipennis*					63	4	14	5							1	1			4	4
*An.plumbeus*							2	2												
*Cx.modestus*	7	3	15	5	97	4	117	12			8	1	2	1						
*Cx.pipiens*	79,543	618	9,437	100	45,905	275	60,96	67	6,512	44	1,629	19	914	43	331	13	158	17	4,528	63
Total	81,346	683	14,924	172	51,456	351	27,439	269	7,105	52	1,652	27	1,176	71	393	27	194	28	4,831	109

See Table 1 for abbreviations of sampling station groups.

The most abundant species resulted *Culex pipiens* (81.4% of total mosquitoes), followed by *Aedes caspius* (15.4%), *Ae. vexans* (2.4%) *Ae. albopictus* (0.6%) and *Cx. modestus* (0.1%). Less abundant species analyzed (under 0.1%) were *Ae. dorsalis, Ae. detritus, Ae. geniculatus, Anopheles maculipennis* s.l., *An. plumbeus* (Table 2).

**Figure 1:**
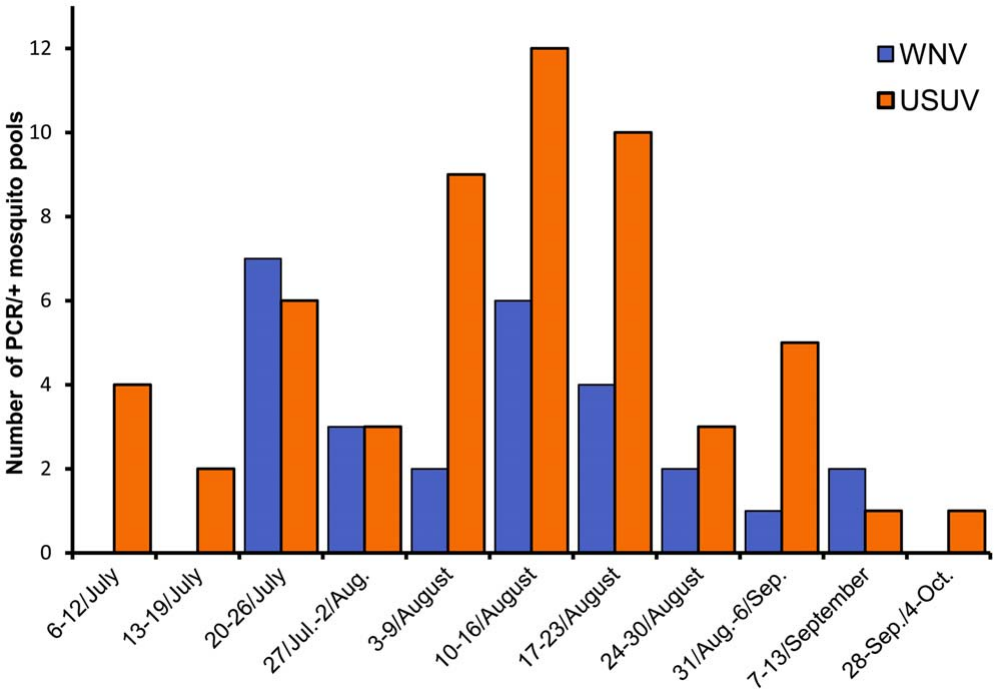
Positive WNV and USUV mosquito pools per week of collection.

**Figure 2:**
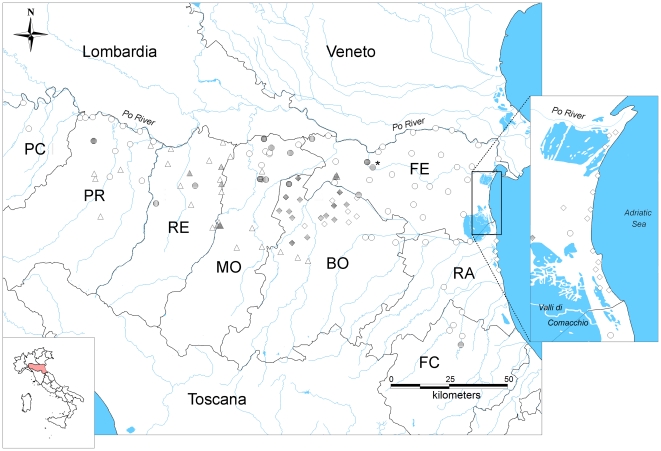
Emilia-Romagna map with the stations included in the survey and locations of PCR–positive pools. Horizontal lines, WNV detections; vertical lines, USUV detections. Diamonds, mosquito abundance monitoring stations; circles, survey stations; triangles, extraordinary stations. Province abbreviations: PC, Piacenza; PR, Parma; RE, Reggio Emilia; MO, Modena; BO, Bologna; RA, Ravenna; FE, Ferrara. (*), the 13 Ferrara city stations were represented with a single circle.

Biomolecular analyses were performed on aliquots of 1,789 mosquito pools (Table 2) giving the subsequent results: 19 WNV-positive pools (sampled in 15 stations), 48 USUV-positive pools (sampled in 24 stations) and 8 positive pools for both viruses (sampled in 7 stations) (Figure 1, Figure 2). Moreover, the WNV was isolated from one pool of *Cx. pipiens* mosquito sampled in Montecchio municipality (Reggio Emilia province) on the August 26. The isolated WNV strain grew with an evident cytopathic effect, until first passage, on Vero cell culture; isolation was confirmed by described ELISA test and by specific reverse transcription-PCR. No isolation was obtained from C6/C36 cell culture.

Additionally 38 mosquito pools were positive for flavivirus-genus PCR only and 26 provided a sequence due to the specific PCR target (part of the *NS5* gene) but not arising from the presence of WNV or USUV. These sequences were detected in 4 mosquito species: 20 in *Ae. albopictus*, 4 in *Ae. caspius*, 1 in *An. maculipennis* s.l. and 1 in *Cx. pipiens*. The 20 sequences obtained from *Ae. albopictus* mosquitoes (GenBank accession numbers: HQ441846, HQ441847, HQ441848, HQ441849, HQ441850, HQ441851, HQ441852, HQ441853, HQ441854, HQ441855, HQ441856, HQ441857, HQ441858, HQ441859, HQ441860, HQ441861, HQ441862, HQ441863, HQ441864, HQ441865) showed an almost complete identity between themselves as well as the 3 sequences from *Ae. caspius* (HQ441843, HQ441844, HQ441845) and that from *Cx. pipiens* (HQ441842); these sequences were very similar with the sequences obtained during the 2007–2008 survey performed in the same area [28] (Figure S1). The sequence obtained from *An. maculipennis* s.l. (HQ441867) and a sequence from *Ae. caspius* (HQ441866) showed an affinity for arthropod-borne flaviviruses, as highlighted in the phylogenetic tree obtained from these sequences and homologous sequences belonging to Flaviviridae family available in Genbank database (Figure S2).

The largest part of mosquitoes was caught by modified CDC traps baited by CO_2_ (CO_2_ traps) [29], 189,258 specimens grouped in 1,724 pools, corresponding to 99.3% of sampled mosquitoes. Only 1,258 mosquitoes (0.7% of the total) were sampled in 14 sites by gravid traps. Gravid traps were only used for sampling in proximity of reported cases of West Nile disease (WND) in horse; collected mosquitoes were grouped in 65 pools, 4 of which were WNV-positive and 2 USUV-positive. Six stations in similar locations were sampled by CO_2_ traps, obtaining 2,579 mosquitoes (grouped in 16 pools) with 4 WNV-positive pools and 9 USUV-positive pools. Collections near WND cases in horse were activated from 1 to 3 weeks after the reporting of case, and captured a total of 8 WNV-positive mosquito pools.

All 27 WNV-positive pools and 54 (out of 56) USUV-positive pools belonged to species *Cx. pipiens*, while two USUV-positive pools belonged to *Ae. albopictus* (Table 2). These two *Ae. albopictus* mosquito pools positive for USUV were sampled on August 17 and 21 in the same municipality.

WNV-positive mosquito pools produced 24 very similar amplicon sequences, part of *E* gene (GenBank accession numbers: HM138724, HM138725, HM138726, HM138727, HM138728, HM138729, HM138730, HM138731, HM138732, HM138733, HM138734, HM138735, HM138736, HM138737, HM138738, HM138739, HM138740, HM138741, HM138742, HM138743, HM138744, HM138745, HM138746, HM138747). The alignment of these sequences, eliminating every gap and missing data, showed an identity ranging from 100 to 99.2% between them, with a number of differences ranging from 0 to 3 on a total of 353 positions considered. Generally, these differences didn't cause modification in consensus deduced aminoacid sequence. In only one sequence a transition (from C to T) in position 545 of retrotranscribed WNV genome (M12294) can lead to a mutation (from threonine to isoleucine) in position 182 of derived translated protein sequence.

The consensus sequence obtained by mosquito pools had a 100% identity with the consensus sequence produced by 21 bird sequences obtained in Emilia-Romagna in 2009 [30] and available in GenBank (data not show), and showed an high identity with other sequences obtained from human (FJ472947, GU011992) and birds (FJ472947, FJ483549, FJ483548) in 2008-09 Italian outbreak. Moreover, this consensus sequence showed a high identity with sequences obtained from mosquito pools sampled in Israel in 2008 (GU246710, GU246708).

Also the 12 partial *NS5* gene USUV sequences, obtained by the flavivirus-genus PCR (GenBank accession numbers HM138707, HM138708, HM138709, HM138710, HM138711, HM138712, HM138713, HM138714, HM138715, HM138716, HM138717, HM138718), showed a good identity, ranged from 100 to 98.3% on 233 positions which were compared, all these differences were silent and did not produce variations in translated amino acid sequence. The highest scores obtained by the BLAST of consensus sequence were with USUV isolated in Budapest (EF206350) and USUV strain Vienna 2001 (AY453411), both showed a 98% identity with the consensus sequence used.

The first pool tested positive for WNV was sampled in Bologna province on July 21 and the last two in Modena Province on September 9 (Figure 1). The highest WNV MLE value in bi-weekly grouped mosquitoes was detected in Reggio Emilia province in August 17–30 (MLE = 2.2 CI = 0.1–13.3). The longest period of circulation was detected in mosquitoes trapped in Modena provinces from July 20 to September 13, where MLE value ranged from 1,1 to 1,9 infected mosquitoes on 1000 tested (Table 4). No WNV-positive pools were sampled in Lidi Ferraresi, Ferrara city, Piacenza and Forlì-Cesena provinces.

**Table 4:** Bi-weekly MLE of WNV and USUV in *Cx. pipiens* mosquito pools for station groups.

	BB	LF	SS	MO	RE	PR
West Nile Virus MLE (95% CI)						
11–24/May	0 (0–1.3)	-	-	-	-	-
25/May–7/Jun.	0 (0–0.2)	0 (0–9.4)	-	-	-	-
8–21/June	0 (0–0.3)	-	0 (0–0.3)	-	-	-
22/Jun–5/Jul	0 (0–0.3)	0 (0–1.6)	0 (0–0.3)	-	0 (0–34)	-
6–19/July	0 (0–0.3)	0 (0–0.8)	0 (0–0.5)	0 (0–23.5)	0 (0–6.4)	-
20/Jul.–2/Aug.	**0.3** (0.1–1)	0 (0–2.2)	**0.7** (0.3–1.5)	**1.9** (0.4–6.4)	0 (0–8.5)	-
3–16/August	**0.4** (0.1–1.4)	0 (0–3.7)	-	**1.7** (0.3–5.8)	0 (0–7.7)	0 (0–1)
17–30/August	**0.7** (0.1–2.2)	0 (0–18.4)	0 (0–1)	0 (0–8.4)	**2.2** (0.1–13.3)	-
31/Aug.–13/Sep.	0 (0–0.9)	0 (0–25.9)	-	**0.6** (0–2.8)	0 (0–13.7)	**0.4** (0.0–1.8)
14–27/September	0 (0–1.1)	-	0 (0–2)	0 (0–2.9)	0 (0–106.5)	-
28-Sep./10-Oct.	0 (0–2.1)	-	0 (0–5.4)	0 (0–7.6)	-	0 (0–6)
Usutu virus MLE (95% CI)						
11–24/May	0 (0–1.3)	-	-	-	-	-
25/May–7/Jun.	0 (0–0.2)	0 (0–9.4)	-	-	-	-
8–21/June	0 (0–0.3)	-	0 (0–0.3)	-	-	-
22/Jun–5/Jul	0 (0–0.3)	0 (0–1.6)	0 (0–0.3)	-	0 (0–34)	-
6–19/July	**0.4** (0.2–1)	0 (0–0.8)	**0.1** (0–0.7)	0 (0–23.5)	0 (0–6.4)	-
20/Jul.–2/Aug.	**1.1** (0.5–2.2)	0 (0–2.2)	**0.3** (0–0.9)	0 (0–2.7)	0 (0–8.5)	-
3–16/August	**1.7** (0.7–3.4)	**1.3** (0.1–7)	-	**2.7** (0.7–7.9)	**2.3** (0.3–16.1)	0 (0–1)
17–30/August	**2.6** (1.1–5.2)	0 (0–18.4)	**0.3** (0–1.4)	0 (0–8.4)	**1.7** (0.1–8.9)	-
31/Aug.–13/Sep.	**0.8** (0.2–2.1)	0 (0–025.9)	-	**0.5** (0–2.7)	0 (0–13.7)	**0.4** (0.0–1.8)
14–27/September	0 (0–1.1)	-	0 (0–2)	0 (0–2.9)	0 (0–106.5)	-
28-Sep./10-Oct.	0 (0–2.1)	-	0 (0–5.4)	0 (0–7.6)	-	0 (0–6)

See Table 1 for abbreviations of sampling station group.

The first 4 pools which tested positive for USUV were sampled in Bologna province (in different sites) on July 8, two weeks before the collection of the first WNV-positive pool, the last one in Forlì-Cesena province on October 3 (Figure 1). The highest USUV MLE value in bi-weekly grouped mosquitoes was detected in Modena province in August 3–16 (MLE = 2.7 CI = 0.7–7.9), the longest period of circulation was detected in Bassa Bolognese samples from July to September, with MLE value ranging from 0.5 to 2.6 (Table 4). No USUV-positive pools were sampled in Piacenza province.

**Figure 3:**
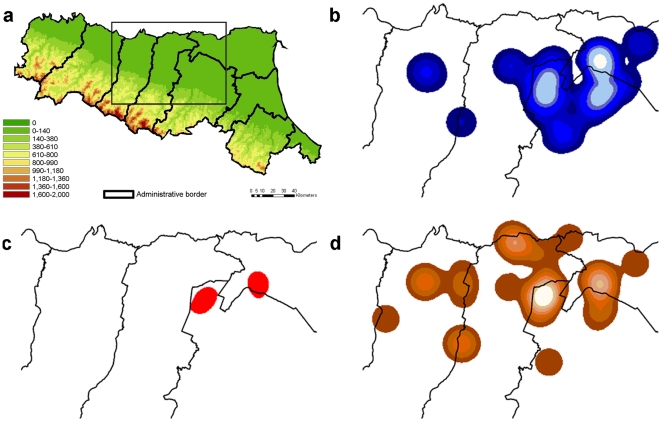
Digital elevation model of Emilia-Romagna region (a) with reference to “hot spots” areas of WNV (b) and USUV (d) obtained by kernel density estimation of positive mosquito pools and overlaying of the two virus dissemination areas with values over the median (c).

The dissemination surfaces of WNV and USUV represented by kernel density estimation (KDE) are represented in Figure 3, the two virus surfaces include different common areas. The overlaying of kernel values over the median of WNV and USUV, produce two areas of strong simultaneous circulation of both viruses, whose centroids are approximately 20 km distant: one in Bologna province (6650 ha) and one in Ferrara province (5456 ha) (Figure 3).

The land use classification showed that 62 traps (39.2%) were placed in urban areas, 31 in green areas (19.6%), 45 in rural areas (28.5%), 10 in both woodlands and wetlands (6.3%).

When Cx. pipiens mosquito pools were classified according to the environment of the sampling stations, most of the pools sampled after the week from July 6 (date of capture of first USUV-positive pool) were from urban areas (414 pools equal of 53.2% of total), 163 pools (21%) were from rural areas, 120 pools (15.4%) were from green area (Table 5).

**Table 5:** Details of pools and PCR positive pools of *Cx pipiens* mosquito collected in the different environments.

	N *Cx. pipiens* pools (%)	N WNV/+ pools (%)	N USUV/+ pools (%)
Urban area	414 (53.2)	8** (29.6)	26 (48.1)
Rural area	163 (21)	14* (51.9)	16 (29.6)
Green area	120 (15.4)	2 (7.4)	8 (14.8)
Wetland	47 (6)	3 (11.1)	4 (7.4)
Woodland	34 (4.4)	0	0
Total	778	27	54

*Statistically significant confront (Cx. pipiens total pools vs. Cx. pipiens WNV-positive pools,

*p<0.01,

**p<0.05)

Generally, the number of USUV-positive pools in different monitored environments reflected the abundance of Cx. pipiens pools in the same environment. Concerning WNV, the proportion of positive pools captured in rural areas was significantly higher (p<0.01), in fact on a total of 778 pools of Cx. pipiens, 163 (21%) were sampled in this environments where 14, out of a total of 27 (51.9%), tested positive for WNV. On the contrary WNV-positive pools were scarce in urban areas (p<0.05), on a total of 414 (53.2%) pools from this environment only 8 (29.6%) were WNV positive. Furthermore WNV-positive pools were frequently observed in wetland (Table 5), even if this evidence wasn't statistically significant. No WNV- and USUV-positive pools were sampled in woodland.

## Discussion

The inquiry conducted on the 190,516 sampled mosquitoes pointed out important epidemiological data on temporal and geographic diffusion of the detected viruses in Emilia-Romagna and their spreads in mosquitoes.

Both WNV and USUV biomolecular data showed a high homology between detected sequences and suggested that a single strain of both WNV and USUV circulated in 2009 in the surveyed area. This hypothesis was confirmed by the high identity also observed between WNV-sequences obtained by mosquito pools and sequences obtained by birds in the same area and available in GenBank. These sequences were also very similar to those obtained the year before in mosquitoes, birds and human, suggesting the virus overwintering between 2008 and 2009. The high identity between sequences obtained and 2008 Israeli sequences suggest a common origin of WNV strains circulating in the two countries in last two years, but further data are necessary to support this observation.

Results of several studies imply a silent circulation of WNV in Europe [18], [21], but the factors triggering WN clinical disease are largely unknown. The abundance of vectors and reservoirs and their distinctive characteristics, like vector competence, vector host preference and host viremia intensity, have a great influence on viral load in the environment; as well as the vector-host interactions, for instance the host availability for vector. Furthermore the interactions of these complex ecological factors are strongly influenced by environmental conditions, such as temperature and rainfall, making the WN outbreak occurrence almost unpredictable. In addition the regular introduction of new virus strains from Mediterranean Asia and Africa [4], [10], [11] could be significant in the amplification of the viral cycle, as the high identity between the obtained WNV sequences and the 2008 Israeli sequences could suggest.

The USUV sequences published on GenBank and containing homologous part of obtained USUV sequences are scarce. Among them the USUV strains originating from Vienna and Budapest showed the highest nucleotide identity rates with the Italian viruses, implying that - following a single introduction of USUV to central Europe - the same USUV strain is now circulating in central Europe.

Twenty-four sequences obtained from positive flavivirus-PCR not referred to WNV or USUV, are due to the presence of RNA of two viruses, also detected during the 2007–2008 survey and probably resulting from the presence of mosquito-only flaviviruses [28]. Moreover two of these sequences are more similar to the arthropod-transmitted flavivirus, and could be a clue of other viruses that are still unknown. These sequence detections in pooled mosquitoes should be taken into consideration in differential diagnosis respect to other flaviviruses.

During 2009, WNV was detected in *Cx. pipiens* pools only, confirming the evidence obtained in 2008 [28], and suggesting this species as the principle vector of WNV in the 2008/2009 outbreak in Northern Italy. WNV wasn't found in other mosquito species abundantly sampled, like *Ae. vexans* and *Ae. caspius*, which leads us to hypothesize the poor involvement of these mosquito species in the virus circulation in Emilia-Romagna. *Cx. modestus* mosquito, which is consider a highly competent species for WNV [10], [31], [32], is scarce in the Po plain, and sampled specimens tested negative for WNV, not showing any evidence of involvement of this species in the epidemic.

All but two positive USUV pools were composed of *Cx. pipiens* mosquito (54 pools), suggesting this species is also the main vector of this virus. Interestingly, USUV was detected in two pools of *Ae. albopictus*, indicating this species as possible USUV vector. Further experimental studies are needed to confirm the vector competence and the role of the Tiger mosquito in the spread of this virus. If this hypothesis will be proved the epidemicity of USUV should be reconsidered, taking into account the uninterrupted spread of Tiger mosquito in the world and the abundance of this invasive species in man-made environments, moreover the health risk connected to the presence of this exotic mosquito will be reconfirmed [33]. Furthermore, this finding could support the ability of *Ae. albopictus* mosquito to serve as a bridge vector, capable of mediating the spillover of a virus from rural-cycle to urban-cycle, role already suggested for this species in regard to other viruses, including WNV [34], [35].

The highest number of mosquitoes analyzed was sampled in regularly working fixed stations (Table 3). The use of extraordinary traps in proximity of reported WND horse cases allowed the capture of 8 WNV-positive pools, confirming the persistence of virus up to 3 weeks in area surrounding horse clinical cases, probably due to the persist of ecological conditions favorable to viral circulation in the environment.

The simultaneous circulation of USUV and WNV in Emilia-Romagna was demonstrated by the presence of positive mosquito pools in the same area and in the same period and also by the presence of mosquito positive pools for both viruses. Furthermore, the KDE analysis produced a largely overlaying area of the two viruses “hot spots” (Figure 3), but this situation could be hidden in scarcely sampled areas. Moreover, as for the WNV [27], the USUV circulation in Northern Italy was not limited to Emilia-Romagna region, as demonstrated by the presence of three *Cx. pipiens* mosquito USUV-positive pools sampled in Lombardia region (Ticino River Park) in August-September 2009 (unpublished data).

Despite the simultaneous circulation and the reported ecological affinity between the two viruses, the data obtained didn't point out any obvious interactions between them. The PCR detections showed a stronger and longer USUV circulation in mosquitoes with respect to WNV, as displayed by the higher number of USUV-positive pools detected and by the presence of USUV-positive pools two weeks earlier than WNV. Indeed the land use data highlighted differences between the ecology of the two viruses. While the USUV-positive pools were uniformly captured in different environments, the number of WNV-positive pools was significantly higher in rural areas, highlighting a greater circulation of this virus in this environment (Table 4). Furthermore USUV was detected in different sampling station groups without WNV-positive pools: Lidi Ferraresi, Ferrara city, Forlì-Cesena province.

Besides the detection in surveyed area of WNV/USUV PCR-positive birds, two European Magpies and one Gull [36], seems to confirm the absence of interaction between the two viruses in vertebrate hosts. These data demonstrated the lack of knowledge of USUV ecology and the need of further experimental investigations to better understand the cycle of this virus.

The earliest evidence of 2009 summer WNV re-activation in Emilia-Romagna region was detected in a mosquito pool sampled in July 21, about a week before the first reported positive bird (July 30) and about three weeks before the six diagnosed WN neuroinvasive human cases reported in second half of August [30]. These results confirmed that, if mosquito trapping effort is intensive, detection of WNV in mosquitoes might precede detection of virus activity by other surveillance tools [37]. Even the first USUV-positive mosquito pools were captured (July 8) before the signaling of human disease case ascribed to this virus, reported approximately from the beginning of September [14], [15].

The spread of the detected viruses in sampled mosquitoes and in different sampled areas were evaluated using MLE, this index could also yield assessment of viral activity under survey epidemiological and ecological conditions. WN MLE data seem to indicate that the virus has resumed the 2008 expansion from east to west (Table 4), along the Po River, like also evidenced by the detection of a WNV mosquito positive pool in Parma province and by the presence of a positive bird captured in Piacenza [30]. USUV MLE data recorded an intensive virus circulation in the central areas of the region (Table 4). Moreover this data on mosquito infection rates provide quantifiable information on intensity of virus transmission and consequently information on potential risk to humans and animals [37]. In this study MLE indexes were calculated by grouping data in homogeneous sets, but the differences in sampling intensity in different areas may influence their values (Table 3). To utilize the mosquito infection rate data to assess the threat of human disease, a network of evenly distributed, regularly working sampling stations is needed over the surveyed area.

The effectiveness of the described survey was demonstrated by the isolation of a WNV strains from a pool of *Cx. pipiens* mosquitoes, but the possibility of virus isolation by the mosquito pool with a PCR positive aliquot can be increased by a better sample management, for instance by limiting freezing and thawing cycles of samples. The introduction of other PCRs in the described monitoring system could allow the detection of other arboviruses, as achieved for Tahyna virus by orthobunyavirus-genus PCR in 2008 [28] and 2009 (unpublished data).

The results obtained by the described survey are encouraging and show how this mosquito-based surveillance could constitute the foundation for a public health alert system targeting mosquito borne arboviruses.

## Materials and Methods

### Survey area

The survey area was the Emilia-Romagna portion of the Pianura Padana, near the Po River, the most important Italian plain, characterized by an intensive agriculture and animal husbandry. The eastern part of the area is located on the Adriatic Sea and it is characterized by the presence of large natural wetland areas (Valli di Comacchio and Po River Delta). The climate condition is typically sub-continental and it becomes gradually Mediterranean toward the costal part of the region. The Pianura Padana environment is strongly influenced by human activities, characterized by intensive agricultural with few hedges, rare scattered trees and a dense irrigation network, non-cultivated zones are rare (eg. beds of rivers, disused quarries). Most common crops are cereals and maize, vineyards or orchards are locally abundant and poplar cultivation is widespread in floodable areas near rivers. The costal part of the region is characterized by pinewood and typical Mediterranean thicket vegetation. All the monitored territories are densely populated and characterized by the abundant presence of villages, city and industrial areas.

### Mosquito collections

Sampling stations utilized in this survey worked with different frequency of sampling. Stations could be clustered by geographic origin and period of sampling into 9 homogenous groups, as shown in Table 1.

Some sampling stations (Bassa Bolognese and Lidi Ferraresi samples) were active since 1991 to monitor mosquito abundance in mosquito control plans, these stations worked weekly for the large part of the mosquito season.

*Other* stations were specifically fixed to monitor the 2008 presumptive WNV circulation area in Ferrara and Bologna provinces; these traps worked monthly and their disposition was set to evenly cover the entire area. Twelve more traps were placed in Modena provinces, and work weekly. Other traps were positioned in Reggio Emilia, Parma, Piacenza and Forlì-Cesena provinces. All these stations were monitored by modified CDC traps baited by CO_2_ (CO_2_ traps) [29].

Moreover extraordinary stations were sampled only once on the regional territory by gravid traps or CO_2_ traps. These traps were activated in proximity of particular epidemiological events, like detection of WNV-positive mosquito pool or following the detection of WND in horses.

All traps were georeferenced and worked at night from roughly 5:00 pm to 9:00 am.

Mosquitoes were identified to species level using morphological characteristics according to three classification keys [38], [39], [40]. *Ochlerotatus* taxon was considered an *Aedes* sub-genus. Mosquitoes were pooled according to date, location and species, with a maximum number of 200 individuals per pool [41], to avoid cross-contamination due to mosquito lose parts, the pools were obtained by handling specimens individually with tweezers. The pooled mosquitoes were stored in polypropylene cryotubes of 2 ml (sample with 100 or smaller) or 4.5 ml, and frozen at −80°. Two 4.3 mm diameter copper plated round balls (Haendler & Natermann Sport GmbH, Münden, D) were added to each 2 ml tube, 4 balls to each 4.5 ml tube. Different amounts of PBS were added in each tube according to the number of stored mosquitoes (0.5 ml until 30 specimens, 0.9 ml from 31 to 60 specimens, 1.5 ml from 61 to 100 specimens and 3 ml from 101 to 200 specimens). Samples were ground for 40 seconds in a vortex mixer, and then centrifuged at 4000 *g* for 3 minutes, finally aliquots were collected from grinded samples and submitted to biomolecular analysis. A 200 µl aliquot was collected by the 101–200 mosquitoes homogenates, to optimize time and costs aliquots from other pools were combined in superpools, with an approach similar to that described in Chisenhall et al. [42]: two 100 µl aliquots from 61–100 specimens homogenates, three 67 µl aliquots from 31–60 specimens homogenates, five 40 µl aliquots from 1–30 specimens homogenates. When a superpool tested positive, each of the original pools that constitute the superpool was tested individually in order to find the specific positive sample.

### Virus survey

RNAs present in aliquots were extracted using Trizol®LS Reagent (Invitrogen, Carlsbad, CA); cDNA synthesis was achieved using random hexamer (Roche Diagnostics, Mannheim, D) and SuperScript® II Reverse transcriptase (Invitrogen, Carlsbad, CA) according to the manufacturer's instructions.

Pools were analyzed using 3 different polymerase chain reactions (PCRs): 1) traditional PCR, targeted to *NS5* gene fragment, for the detection of flavivirus-genus according to Scaramozzino et al. [43], 2) traditional PCR for the detection of USUV [44] and 3) real time PCR for the detection of part of *E* gene of WNV according to the method of Tang et al. [45].

Fragments obtained by flavivirus-genus PCR were sequenced by an automated fluorescence-based technique following the manufacturer's instructions (ABI-PRISM 3130 Genetic Analyzer, Applied Biosystems, Foster City, CA). In order to obtain *E*-gene sequences of detected WNV a traditional PCR protocol was performed on positive samples with the same primers as the real time-PCR protocol.

These sequences were employed to perform basic local alignment search tool (BLAST) in the GenBank library to confirm the specificity of positive reaction and to estimate the degree of identity of detected strains. The sequences obtained were aligned with available GenBank sequences by ClustalW of the freeware program MEGA 4 [46].

Virus isolation was attempted starting from the remaining part of PCR positive mosquito homogenates using Vero cell line (American Type Culture Collection, Rockville, MD), incubated at 37°C, and C6/36 cell line [47] at 28°C.

For WNV and USUV identification, supernatant fluids of inoculated cell cultures were tested through two different sandwich enzyme-linked immunosorbent assay (ELISA). The first ELISA, specific for WNV, was performed by using a monoclonal antibody reactive against WNV envelope protein domain III (EDIII) while the other sandwich-ELISA, was performed using monoclonal antibodies cross reactive between members of Japanese encephalitis antigenic complex [48]. Furthermore supernatant from cell cultures showing cytopathic effects were submitted to the described specific reverse transcription-PCR.

### Statistical analysis and point pattern analysis

To estimate the proportion of infected mosquitoes in *Cx. pipiens* samples maximal likelihood estimation (MLE) was calculated [49] by the Poolscreen2 program [50]. The data were analyzed by grouping together *Cx. pipiens* pools sampled in two weeks in homogeneous groups of sites, with at least 1,500 sampled specimens throughout the season.

Land use characteristics of the sampling stations were obtained by overlaying georeferenced traps to Emilia-Romagna Region Land Use 2003 layer in ArcView 3.x GIS. According to obtained data, stations were ranked in five categories: urban area (residential area, commercial/industrial area), rural area (arable, farm), green area (gardens, urban parks, sport facilities), woodland (natural/artificial woods, orchards), wetland (swamps, marshes, quarries, river bank).

Abundance of positive PCR pools and total number of Cx. pipiens pools sampled in different environment were compared by using χ^2^ test (p<0.05).

Kernel density estimation (KDE) was applied on two datasets composed by the mosquito pools positive for WNV and USUV. KDE is a geospatial technique based on the kernel function, a bivariate probably density function [51], it is used to create a surface to indicate the intensity of the events of the phenomenon. KDE was used as exploratory analysis for identifying possible “hot spots” indicating the spatial clusters of the presence of WNV an USUV. The bandwidth size of 10 km was taken to prevent the formation of spatially discrete patterns or surfaces (too small bandwidth) or smoother surfaces of the derived intensity (too large bandwidth) [52]. To evaluate areas with an intensive and comparable simultaneous circulation of both viruses, KDE surfaces of each virus, with kernels value above the median, were overlapped. KDE were performed using ESRI ArcView 3.x GIS and ArcView Spatial Analyst Extension.

## Supporting Information

Figure S1 Phylogenetic trees of the sequences of detected mosquito-only flaviviruses, other mosquito-only flaviviruses and yellow fever virus. The Neighbor-Joining Phylogeny tree of a portion of NS5 gene, amplified by flavivirus-PCR, was constructed by the informatics program MEGA4 (model p-distance) with the sequences of amplified fragments and homologous fragment of flaviviruses obtained in GenBank library. Only the percentages over 70% in the bootstrap test (1000 replicates) are shown next to the branches. ABBREVIATIONS (GenBank accession number); study area detected sequences: AealFV2: sequence detected in *Aedes albopictus* in 2008 (GQ477006), FE-Aa1: sequence detected in *Ae. albopictus* in 2009 (HQ441847), AeveFV2: sequence detected in *Ae. vexans* in 2008 (GQ477001), OccaFV3: sequence detected in *Ae. caspius* in 2007 (GQ476995), OccaFV4: sequence detected in *Ae. caspius* in 2008 (GQ476991), FE-Cp: sequence detected in *Culex pipiens* in 2009 (HQ441842), FE-Ac3: sequence detected in *Ae. caspius* in 2009 (HQ441845); GenBank sequences: AeFV: *Aedes* flavivirus (AB488408), CxFV: *Culex* flavivirus (Tokyo: AB262759, Mex: EU879060; USA: FJ502995), CFA: cell fusing agent (NC001564; Culebra: DQ181514; Rio Piedras: EU074056), KRV: Kamiti River virus (SR-82: AY149905, SR-75: AY149904), PoMoFlavA153: sequence detected in *Ae. caspius* sampled in Portugal in 2007 (EU716422), YFV: Yellow fever virus (U54798).
(0.46 MB TIF)

Figure S2 Phylogenetic trees of the sequences of presumptive detected flaviviruses and other viruses of family *Flaviviridae*. The Neighbor-Joining Phylogeny tree of a portion of NS5 gene, amplified by flavivirus-PCR, were constructed by the informatics program MEGA4 (model p-distance) with the sequences of amplified fragments and homologous fragment of *Flaviviridae* obtained in GenBank library. *Flavivirus* genus branch was colored in black, *Pestivirus* genus was in azure and *Hepacivirus* genus was in blue. *Flavivirus* genus was further divided into insect-only viruses in green, non-know vector in orange, tick-borne in yellow and mosquito-borne in red. ABBREVIATIONS (GenBank accession number); study area detected sequences: MO-Ac: sequence detected in *Aedes caspius* in 2009 (HQ441866), PV-Am: sequence detected in *Anopheles maculipennis* in 2009 (HQ441867), OccaFV3: sequence detected in *Ae. caspius* in 2007 (GQ476995). GenBank sequences: AeFV: *Aedes* flavivirus (AB488408), APOIV: Apoi virus (NC003676), BORV: Border disease virus (NC003679), BVDV: Bovine viral diarrhea virus (NC001461), CFA: cell fusing agent (NC001564), CSFV: Classical swine fever virus (NC002657), CxFV: *Culex* flavivirus (AB262759), DENV: Dengue virus (1: U88536, 2: NC001474, 3: NC001475, 4: NC002640), HCV: Hepatitis C virus (1: AF009606, 2: AY746460, 3: GU814263), JEV: Japanese encephalitis virus GQ902063, KRV: Kamiti River virus (AY149905), MODV: Modoc virus (NC003635), OHFV: Omsk hemorragic fever virus (NC005062), POWV: Powassan virus (NC003687), RBV: Rio bravo virus (NC003675), TBEV: Tick-borne encephalitis virus (DQ401140), USUV: Usutu virus (HM138707), WNV: West Nile virus (GU047875), YFV: Yellow fever virus (U54798).
(1.86 MB TIF)
