# Supplementary figures and images for: Evidence of Simultaneous Circulation of West Nile and Usutu Viruses in Mosquitoes Sampled in Emilia-Romagna Region (Italy) in 2009
Source: PLoS One. 2010 Dec 15;5(12):e14324. doi: 10.1371/journal.pone.0014324 (PMC3002278; doi:10.1371/journal.pone.0014324)

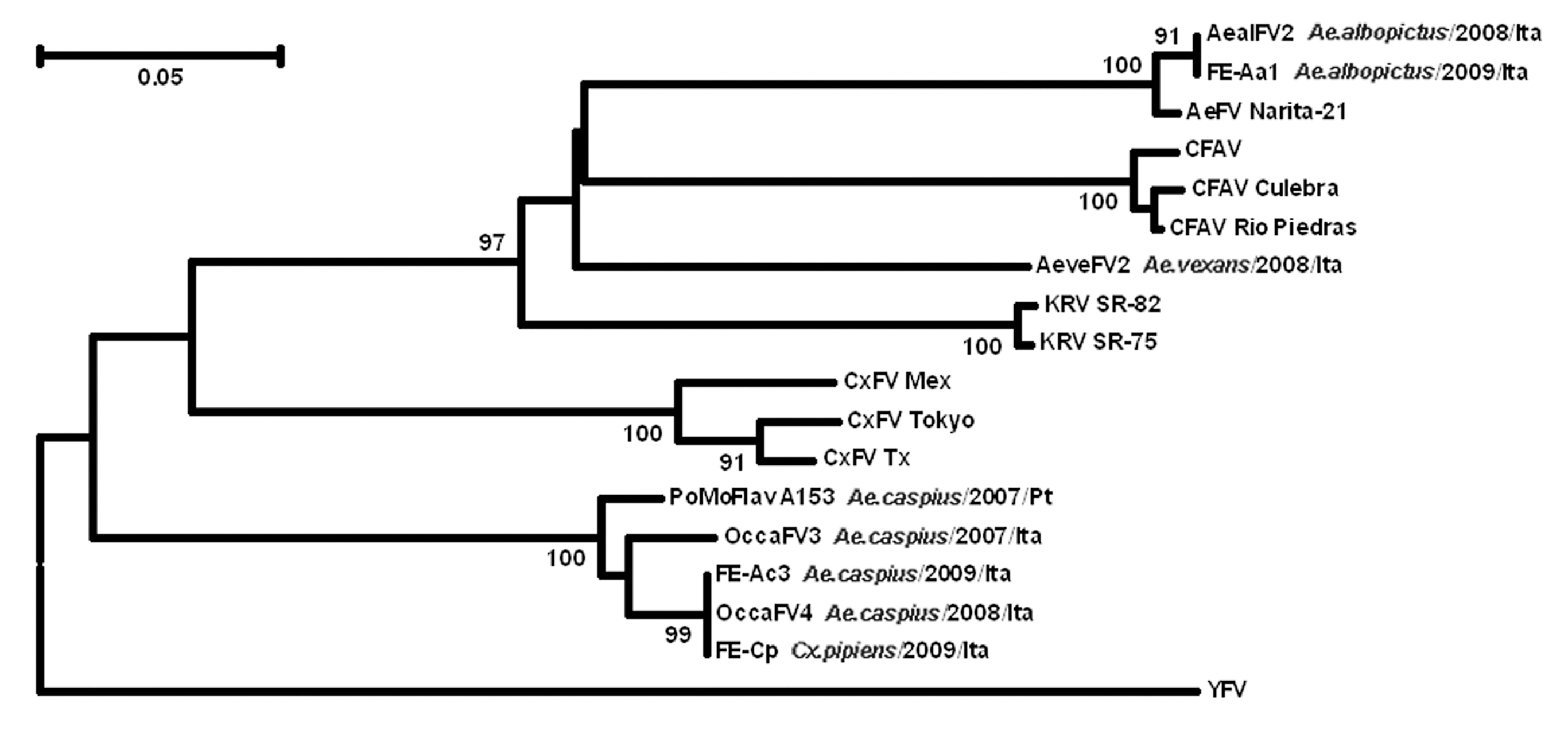

Supplement: Figure S1 — Phylogenetic trees of the sequences of detected mosquito-only flaviviruses, other mosquito-only flaviviruses and yellow fever virus. The Neighbor-Joining Phylogeny tree of a portion of NS5 gene, amplified by flavivirus-PCR, was constructed by the informatics program MEGA4 (model p-distance) with the sequences of amplified fragments and homologous fragment of flaviviruses obtained in GenBank library. Only the percentages over 70% in the bootstrap test (1000 replicates) are shown next to the branches. ABBREVIATIONS (GenBank accession number); study area detected sequences: AealFV2: sequence detected in Aedes albopictus in 2008 (GQ477006), FE-Aa1: sequence detected in Ae. albopictus in 2009 (HQ441847), AeveFV2: sequence detected in Ae. vexans in 2008 (GQ477001), OccaFV3: sequence detected in Ae. caspius in 2007 (GQ476995), OccaFV4: sequence detected in Ae. caspius in 2008 (GQ476991), FE-Cp: sequence detected in Culex pipiens in 2009 (HQ441842), FE-Ac3: sequence detected in Ae. caspius in 2009 (HQ441845); GenBank sequences: AeFV: Aedes flavivirus (AB488408), CxFV: Culex flavivirus (Tokyo: AB262759, Mex: EU879060; USA: FJ502995), CFA: cell fusing agent (NC001564; Culebra: DQ181514; Rio Piedras: EU074056), KRV: Kamiti River virus (SR-82: AY149905, SR-75: AY149904), PoMoFlavA153: sequence detected in Ae. caspius sampled in Portugal in 2007 (EU716422), YFV: Yellow fever virus (U54798). (0.46 MB TIF) [file pone.0014324.s001.tif]

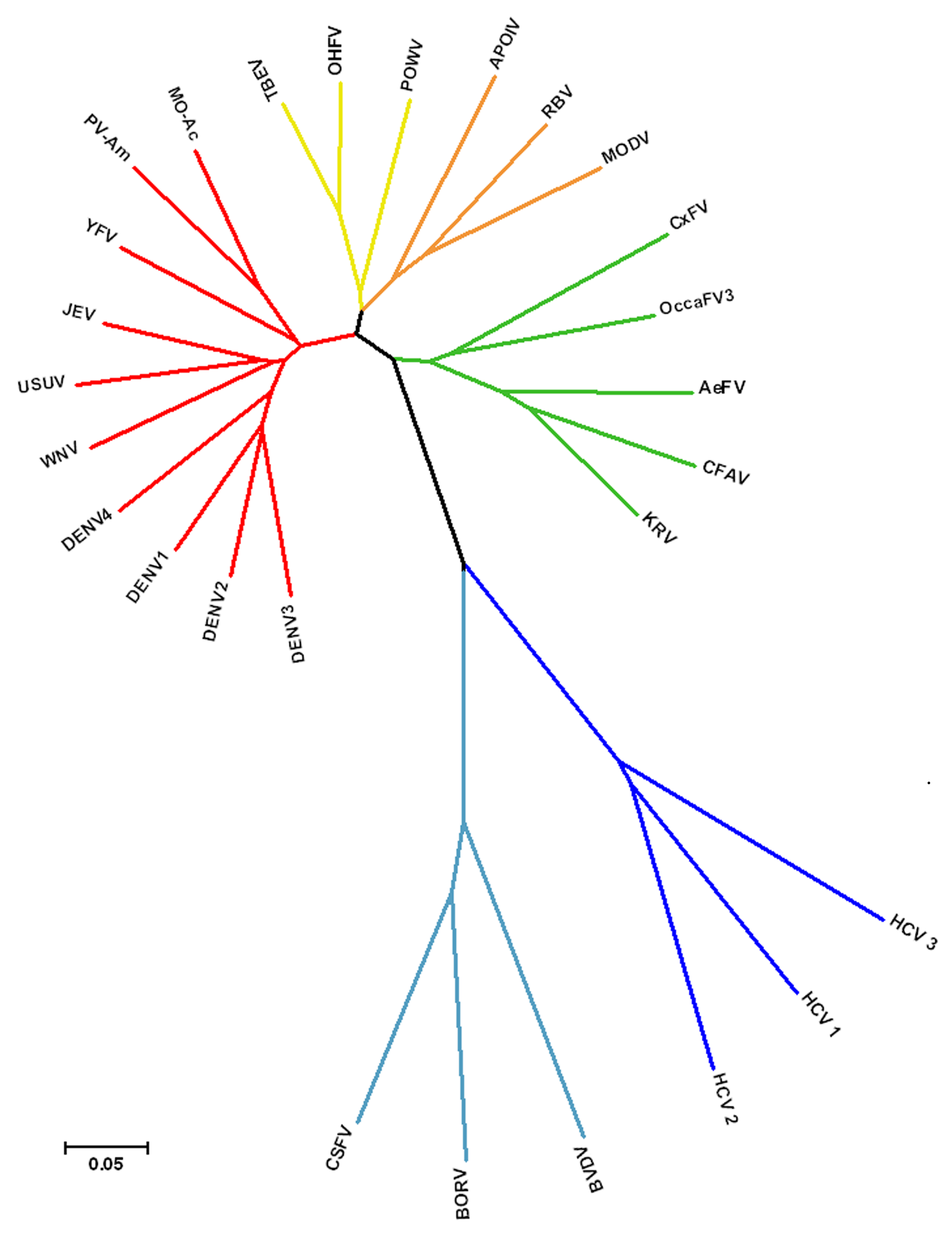

Supplement: Figure S2 — Phylogenetic trees of the sequences of presumptive detected flaviviruses and other viruses of family Flaviviridae. The Neighbor-Joining Phylogeny tree of a portion of NS5 gene, amplified by flavivirus-PCR, were constructed by the informatics program MEGA4 (model p-distance) with the sequences of amplified fragments and homologous fragment of Flaviviridae obtained in GenBank library. Flavivirus genus branch was colored in black, Pestivirus genus was in azure and Hepacivirus genus was in blue. Flavivirus genus was further divided into insect-only viruses in green, non-know vector in orange, tick-borne in yellow and mosquito-borne in red. ABBREVIATIONS (GenBank accession number); study area detected sequences: MO-Ac: sequence detected in Aedes caspius in 2009 (HQ441866), PV-Am: sequence detected in Anopheles maculipennis in 2009 (HQ441867), OccaFV3: sequence detected in Ae. caspius in 2007 (GQ476995). GenBank sequences: AeFV: Aedes flavivirus (AB488408), APOIV: Apoi virus (NC003676), BORV: Border disease virus (NC003679), BVDV: Bovine viral diarrhea virus (NC001461), CFA: cell fusing agent (NC001564), CSFV: Classical swine fever virus (NC002657), CxFV: Culex flavivirus (AB262759), DENV: Dengue virus (1: U88536, 2: NC001474, 3: NC001475, 4: NC002640), HCV: Hepatitis C virus (1: AF009606, 2: AY746460, 3: GU814263), JEV: Japanese encephalitis virus GQ902063, KRV: Kamiti River virus (AY149905), MODV: Modoc virus (NC003635), OHFV: Omsk hemorragic fever virus (NC005062), POWV: Powassan virus (NC003687), RBV: Rio bravo virus (NC003675), TBEV: Tick-borne encephalitis virus (DQ401140), USUV: Usutu virus (HM138707), WNV: West Nile virus (GU047875), YFV: Yellow fever virus (U54798). (1.86 MB TIF) [file pone.0014324.s002.tif]
